# Health Care Providers’ and Professionals’ Experiences With Telehealth Oncology Implementation During the COVID-19 Pandemic: A Qualitative Study

**DOI:** 10.2196/29635

**Published:** 2022-01-19

**Authors:** Kea Turner, Margarita Bobonis Babilonia, Cristina Naso, Oliver Nguyen, Brian D Gonzalez, Laura B Oswald, Edmondo Robinson, Jennifer Elston Lafata, Robert J Ferguson, Amir Alishahi Tabriz, Krupal B Patel, Julie Hallanger-Johnson, Nasrin Aldawoodi, Young-Rock Hong, Heather S L Jim, Philippe E Spiess

**Affiliations:** 1 Department of Health Outcomes and Behavior Moffitt Cancer Center Tampa, FL United States; 2 Department of Supportive Care Medicine Moffitt Cancer Center Tampa, FL United States; 3 Virtual Health Program Moffitt Cancer Center Tampa, FL United States; 4 Department of Health Outcomes and Biomedical Information University of Florida Gainesville, FL United States; 5 Department of Internal and Hospital Medicine Moffitt Cancer Center Tampa, FL United States; 6 Division of Pharmaceutical Outcomes and Policy University of North Carolina at Chapel Hill UNC Lineberger Comprehensive Cancer Center Chapel Hill, NC United States; 7 Department of Medicine University of Pittsburgh Pittsburgh, PA United States; 8 Department of Head and Neck-Endocrine Oncology Moffitt Cancer Center Tampa, FL United States; 9 Department of Anesthesiology Moffitt Cancer Center Tampa, FL United States; 10 Department of Health Services Research, Management and Policy University of Florida Tampa, FL United States; 11 Department of Genitourinary Oncology Moffitt Cancer Center Tampa, FL United States

**Keywords:** telehealth, telemedicine, teleoncology, digital health, remote monitoring, cancer, oncology, coronavirus disease, COVID-19

## Abstract

**Background:**

Rapid implementation of telehealth for cancer care during COVID-19 required innovative and adaptive solutions among oncology health care providers and professionals (HPPs).

**Objective:**

The aim of this qualitative study was to explore oncology HPPs’ experiences with telehealth implementation during the COVID-19 pandemic.

**Methods:**

This study was conducted at Moffitt Cancer Center (Moffitt), an NCI (National Cancer Institute)-Designated Comprehensive Cancer Center. Prior to COVID-19, Moffitt piloted telehealth visits on a limited basis. After COVID-19, Moffitt rapidly expanded telehealth visits. Telehealth visits included real-time videoconferencing between HPPs and patients and virtual check-ins (ie, brief communication with an HPP by telephone only). We conducted semistructured interviews with 40 oncology HPPs who implemented telehealth during COVID-19. The interviews were recorded, transcribed verbatim, and analyzed for themes using Dedoose software (version 4.12).

**Results:**

Approximately half of the 40 participants were physicians (n=22, 55%), and one-quarter of the participants were advanced practice providers (n=10, 25%). Other participants included social workers (n=3, 8%), psychologists (n=2, 5%), dieticians (n=2, 5%), and a pharmacist (n=1, 3%). Five key themes were identified: (1) establishing and maintaining patient-HPP relationships, (2) coordinating care with other HPPs and informal caregivers, (3) adapting in-person assessments for telehealth, (4) developing workflows and allocating resources, and (5) future recommendations. Participants described innovative strategies for implementing telehealth, such as coordinating interdisciplinary visits with multiple HPPs and inviting informal caregivers (eg, spouse) to participate in telehealth visits. Health care workers discussed key challenges, such as workflow integration, lack of physical exam and biometric data, and overcoming the digital divide (eg, telehealth accessibility among patients with communication-related disabilities). Participants recommended policy advocacy to support telehealth (eg, medical licensure policies) and monitoring how telehealth affects patient outcomes and health care delivery.

**Conclusions:**

To support telehealth growth, implementation strategies are needed to ensure that HPPs and patients have the tools necessary to effectively engage in telehealth. At the same time, cancer care organizations will need to engage in advocacy to ensure that policies are supportive of oncology telehealth and develop systems to monitor the impact of telehealth on patient outcomes, health care quality, costs, and equity.

## Introduction

The onset of the COVID-19 pandemic in March 2020 accelerated the rapid adoption of telehealth, which is health care delivery at a distance [1-4]. Prior to the COVID-19 pandemic, health care systems faced substantial barriers to telehealth adoption, such as limited reimbursement and start-up hurdles (eg, infrastructure costs) [5-9]. In the United States, telehealth had spread in some health care markets (eg, neurology) due to policy reform, while diffusion into other markets, such as oncology, was minimal [10-13]. Widespread federal and state changes to telehealth regulation, payment, and insurance benefit design as a result of COVID-19 reduced many entry barriers, allowing health care systems to deploy telehealth for oncology [4,14-16]. This seismic shift in telehealth policy created a window of opportunity to redesign cancer care for a virtual setting. In response, cancer care systems invested in large-scale organizational change, such as adopting new technologies and changing billing processes, to support telehealth implementation [17-21]. Much of this work was led by cancer care frontline workers who quickly adapted their practice to a virtual environment. The 1-year anniversary of COVID-19 was an opportunity to evaluate oncology care teams’ experiences with telehealth and assess the role that telehealth can play in cancer care in the future.

Before the pandemic, the Centers for Medicare & Medicaid Services (CMS) defined telehealth as real-time videoconferencing between health care providers and professionals (HPPs) and patients [22]. During the pandemic, CMS broadened its definition of telehealth to include virtual check-ins (ie, brief communication with a practitioner by telephone or another form of telecommunication) and e-visits (ie, asynchronous communication between patients and HPPs through a secure platform, such as a patient portal). In response, oncology care teams began to implement telehealth in various forms [17-21]. In the span of just a few weeks after the pandemic hit, many cancer care systems implemented telehealth for the first time and rapidly scaled virtual care to more than half of their patient population [18-20]. HPPs were required to adapt elements of the physical exam to a telehealth visit and maintain strong relationships with patients from a distance [17,20,21,23-25]. HPPs were also faced with a long-standing digital divide, that is, disparities in patients’ technology know-how and access [26]. Early reports of telehealth implementation described challenges, such as limited device access, lack of home broadband, limited digital literacy, and lack of technology education programs for patients [26]. Further, HPPs were required to make complex decisions around patient care, such as determining which conditions and patients are appropriate for virtual care [17,24].

To capture experiences with telehealth implementation during COVID-19, we conducted a qualitative study with HPPs at a cancer center. We discuss insights from oncology HPPs regarding the successes and challenges with telehealth implementation and recommendations for future telehealth delivery.

## Methods

### Setting, Recruitment, and Eligibility Criteria

This study was conducted at Moffitt Cancer Center (Moffitt), an NCI (National Cancer Institute)-Designated Comprehensive Cancer Center, located in Tampa, Florida. Prior to COVID-19, Moffitt piloted telehealth visits (ie, real-time videoconferencing between HPPs and patients) [22] on a limited basis. After the start of the COVID-19 pandemic, Moffitt rapidly expanded telehealth visits; the volume increased by 5000% from pre–COVID-19 to after the start of the COVID-19 pandemic [27]. Moffitt also instituted virtual check-ins (ie, brief communication with an HPP by telephone only) [22] as a part of telehealth delivery. Moffitt did not employ any other telecommunication technologies for virtual check-ins (eg, secure text messages) and did not implement any other forms of telehealth (eg, e-visits). The Moffitt Virtual Health Program—a centralized support system for the institution—provided information technology (IT) support for patients and HPPs, technical assistance for HPPs (eg, help with out-of-state licensure questions), and an interpreter service for patients with limited English proficiency. Telehealth visits were implemented through Zoom software (Zoom Video Communications, Inc), which was not integrated into Moffitt’s electronic health record (EHR) system. The telehealth visits were conducted in various locations depending on what space was available to the HPP (eg, private office, shared office, and conference room). Some HPPs also elected to deliver telehealth visits from their home.

The target population included all full-time HPPs in oncology at Moffitt. HPPs were defined as health care providers who are licensed to diagnose and deliver treatment (eg, physicians, advanced practice providers [APPs], and psychologists) and professionals who are licensed to deliver other health services (eg, dieticians, social workers, and pharmacists). Oncology was defined, using the NCI’s definition, as a field of medicine concerned with the diagnosis and treatment of cancer [28]. We excluded trainees (eg, fellows, residents, and interns), given that their experience with adapting to telehealth would likely be distinct from HPPs. Since trainees are new to delivering care, we opted for interviewing HPPs who had experience with delivering care prior to the pandemic and may be better positioned to describe how health care had changed because of the pandemic. For recruitment, we sent recruitment emails to two institution-wide listservs on July 1, 2021; presented the study at a leadership meeting that included all department chairs on July 15, 2021; and asked participating HPPs to suggest additional participants. We recruited until no new themes emerged or when data saturation was achieved [29].

### Data Collection

The research team developed a semistructured interview guide to assess oncology HPPs’ experiences with telehealth during COVID-19 (Multimedia Appendix 1). The interviews assessed how HPPs were using telehealth, adaptations necessary to shift in-person care to virtual, and perceived successes, challenges, and lessons learned. The interview guide was developed based on the Consolidated Framework for Implementation Research (CFIR) that identifies common, multilevel barriers and facilitators to implementing new programs, such as leadership support, resources, and individuals’ knowledge about the intervention [30]. The guide was structured around the five CFIR domains and focused on constructs most relevant to telehealth implementation, including the following: (1) intervention characteristics (eg, ease of implementation of telehealth platform), (2) inner setting characteristics (eg, access to information about the intervention), (3) outer setting characteristics (eg, reimbursement and licensure policies related to telehealth), (4) individual characteristics (eg, patient and HPP knowledge about telehealth), and (5) process (eg, planning for telehealth implementation).

Participants were also asked about the future of oncology telehealth and whether they were supportive of using telehealth beyond COVID-19. An individual trained in qualitative methods conducted 40 individual interviews from August to December 2020 with HPPs via Zoom videoconference. An audio recording was generated through QuickTime Player (Apple Inc). We limited the interviews to 30 minutes to ensure that HPPs could participate. To stay close to the 30-minute time frame, we alerted all participants when 25 minutes had passed, assessed how many questions were left at the 25-minute mark, and if more than one question was left, we asked participants if they could stay on for a few additional minutes to finish all the interview questions. We were able to ask all participants all the interview questions using this approach. Participants provided informed consent via videoconference. Participants were asked to provide oral consent for interview participation and then asked to provide oral consent to having the interview recorded. The interviews were recorded and transcribed verbatim by a professional transcription service (GMR Transcription Services, Inc). The transcripts were deidentified (ie, all identifying information about participants was removed) and were assigned a participant ID. The Advarra Institutional Review Board reviewed and exempted this study. Participants were not compensated for their participation.

### Data Analyses

We applied a hybrid approach of integrating deductive and inductive coding, which is commonly used in implementation science research and allows researchers to use theory-driven and data-driven coding [31,32]. We developed a codebook based on deductive codes [33] from the interview guide and inductive codes [34] generated from themes within the data. The interviews were coded by two independent coders using Dedoose software (version 4.12). The coders applied codes to an initial set of transcripts (n=5) independently, compared and refined coding until consensus was achieved, and finished coding the remaining transcripts. A high level of interrater reliability (κ=0.81) was achieved. The κ coefficient was defined as the number of characters coded and not coded by both coders (ie, agreement) divided by the total number of characters [35]. A summary of key themes was sent to 5 participants to confirm that the researchers’ interpretation of the data was consistent with participants’ experiences (ie, member checking) [29]. We reported on themes that were discussed by a minimum of 10 participants to ensure that we were summarizing central themes. Within themes, we noted when there was consensus or divergence across HPPs. The findings are reported based on the Consolidated Criteria for Reporting Qualitative Studies checklist [36].

## Results

### Participant Characteristics

Approximately half of the 40 participants were physicians (n=22, 55%), and one-quarter of the participants were APPs (n=10, 25%; Table 1). Other participants included social workers (n=3, 8%), psychologists (n=2, 5%), dieticians (n=2, 5%), and a pharmacist (n=1, 3%). Most participants were female (n=24, 60%). Participants represented 17 clinical areas. Few participants (n=6, 15%) had used telehealth prior to COVID-19, but all were using telehealth during COVID-19. Participants used telehealth across the cancer care continuum, including screening, diagnosis and follow-up, surveillance, supportive care, procedure preparation and follow-up, and survivorship care.

**Table 1:** Participant characteristics.

Characteristics		Value (N=40)
**Job role, n (%)^a^**		
	Advanced practice provider^b^	10 (25)
	Dietician	2 (5)
	Pharmacist	1 (3)
	Physician^c^	22 (55)
	Psychologist	2 (5)
	Social worker	3 (8)
**Gender, n (%)**		
	Female	24 (60)
	Male	16 (40)
**Clinical focus, n (%)**		
	Breast oncology	2 (5)
	Bone marrow transplant	2 (5)
	Cutaneous oncology	2 (5)
	Endocrinology	1 (3)
	Gastrointestinal oncology	4 (10)
	Genitourinary oncology	3 (8)
	Gynecologic oncology	2 (5)
	Head and neck cancer	2 (5)
	Interventional radiology	2 (5)
	Neuro-oncology	1 (3)
	Radiation oncology	2 (5)
	Sarcoma	3 (8)
	Senior adult	1 (3)
	Social work	3 (8)
	Supportive care	4 (10)
	Survivorship clinic	2 (5)
	Thoracic oncology	4 (10)
**Clinical affiliation, n (%)**		
	Single-site practice	37 (93)
	Multisite practice	3 (8)
Job tenure (years), mean (SD)		12.5 (6.9)
**Virtual visits pre–COVID-19, n (%)**		
	Yes	6 (15)
	No	34 (85)
**Virtual visits during COVID-19, n (%)**		
	Yes	40 (100)
	No	0 (0)

^a^Some of the percentages may not add to 100 due to rounding.

^b^Advanced practice providers included nurse practitioners and physician assistants.

^c^Physicians included endocrinologists, medical oncologists, palliative care specialists, psychiatrists, radiation oncologists, radiologists, and surgeons.

### Qualitative Analysis

Five key themes were identified: (1) establishing and maintaining patient-HPP relationships, (2) coordinating care with other HPPs and informal caregivers, (3) adapting in-person assessments for telehealth, (4) developing workflows and allocating resources, and (5) future recommendations. For each theme, the codes, code definitions, and frequency of use across participants are presented in tables in the following sections, while illustrative quotations are presented in the sections following the tables.

### Theme 1: Establishing and Maintaining Patient-HPP Relationships

#### Overview

Participants described how telehealth changed the patient-HPP relationship, including information exchange, patient engagement, and emotional response (Table 2).

**Table 2:** Theme 1: establishing and maintaining patient-HPP relationships codebook.

Parent code and child codes		Code definition	Code frequency across participants (N=40), n (%)
**Patient-HPP^a^ communication**			
	Patient receptivity to information	Apply code when participant discusses how patient-HPP communication is easier due to patient’s increased receptivity to receive information in their home environment.	21 (53)
	Easier to share screen with patient to display results	Apply code when participant discusses how it is easier to share screen and display results with a patient during telehealth visits.	12 (30)
	Patient-initiated discussion and questions	Apply code when participant describes how telehealth visits affect patients’ willingness to initiate discussion about condition or ask questions.	11 (28)
**Patient-HPP engagement**			
	Requires more energy from the HPP	Apply code when participant describes having to put on an act, be more dynamic, or put more energy into telehealth visits to engage patients.	27 (68)
	Value of video	Apply code when participant describes how the video component of telehealth visits is important for patient-HPP engagement.	10 (25)
	Lack of physical connection	Apply code when participant describes how lack of physical connection (ie, ability to touch patient) affects the delivery of medicine through telehealth.	12 (30)
	Communicating difficult news	Apply code when participant describes the challenges of delivering difficult news through telehealth (eg, new and serious diagnosis).	23 (58)

^a^HPP: health care provider and professional.

#### Information Exchange

Participants felt that patient education was easier to deliver virtually because patients were more relaxed at home compared to the clinic and may be more receptive to receiving information. For example, one dietician explained as follows:

Because they’re typically at home, they’re more relaxed. They’re not in this clinic environment. So, it’s almost like they’re a little bit more receptive to what you’re saying because they’re home in their own comfortable environment.

#### Patient Engagement

In contrast, some participants felt that patients were more reluctant to speak up and ask questions during telehealth visits compared to in-person visits, making information exchange more difficult. One APP described this as follows:

I think the connection, it’s not always there. It makes it a little harder to have that quick back-and-forth dialogue. When you’re trying to get a lot of information across, I feel like patients are listening more than they are having a conversation back with you.

Participants noted that patient engagement was more challenging during telehealth visits. Participants described how telehealth visits required more energy from HPPs. One APP said the following:

When I’m talking to patients over Zoom, those are really shorter visits. There’s not as much discussion over Zoom. I fear that we’re gonna miss some things because of it. I try to slow down a little bit during the Zoom visit and really try to hold their attention a little bit longer. It requires more focus on my part than an in-person visit.

Participants also noted that engagement was harder during virtual check-ins delivered by phone compared to telehealth visits with video. One social worker indicated the following:

Some people they’re not using their picture or they’re calling in, so you don’t know what’s going on. So, something does get lost.

#### Emotional Response

Some participants also found it more challenging to respond to patients’ emotions during telehealth visits. One physician described the following:

I think sometimes it’s nice when you can hold somebody’s hand or give them a hug. Obviously, now, the COVID world, you know, we’re not able to do that as much. But especially through a screen when you’re not there in person, you lose a bit of that connection.

As a result, some HPPs felt that certain tasks, such as delivering difficult news, are better suited for in-person visits. For example, one APP shared the following:

This is a new diagnosis. They should be in the clinic so that I can emotionally support them, offer other services while they're in here—if they need to talk to the social worker. Otherwise, they may hang up, and it may be too devastating, and we may not be able to connect again.

### Theme 2: Coordinating Care With Other HPPs and Informal Caregivers

#### Overview

Participants indicated that telehealth made it easier to coordinate care with other HPPs and informal caregivers, such as family members or friends (Table 3). This theme was primarily discussed by health care providers (eg, physicians and APPs) as opposed to other health care professionals (eg, dieticians).

**Table 3:** Theme 2: care coordination with other HPPs and informal caregivers codebook.

Parent code and child codes			Code definition	Code frequency across participants (N=40), n (%)	
**HPP^a^-HPP coordination**					
	Coordinating with external HPPs	Apply code when participant describes coordinating telehealth visits with health care HPPs external to Moffitt^b^.			18 (45)
	Coordinating with internal HPPs	Apply code when participant describes coordinating telehealth visits with health care HPPs internal to Moffitt (eg, other specialties).			22 (55)
**HPP-caregiver coordination**					
	Allowing caregivers to join in-person visits through telehealth	Apply code when participant describes using Zoom or other platform to allow caregiver to participant in a patient’s in-person visit.			30 (75)
	Allowing caregivers to join patients’ telehealth visits	Apply code when participant describes including caregivers in patient’s telehealth visit.			25 (63)

^a^HPP: health care provider and professional.

^b^Moffitt: Moffitt Cancer Center.

#### Care Coordination With HPPs

Participants discussed how telehealth made it possible to bring together HPPs within the same institution. One physician recalled the following:

I’ve had one of the surgeons, myself, and the medical oncologist, and the radiation oncologist, and the patient all on Zoom at one time, so true multidisciplinary care provided through the Zoom platform. I’ve done that in one instance where the patient was in the room with me and I was doing the physical exam and we Zoomed-in the medical oncologist as part of a kind of multidisciplinary assessment.

Participants also described how telehealth visits made it much easier to coordinate care with HPPs outside of Moffitt. For example, one physician described the following:

I’ve got a few patients that I share with oncologists in other parts of the country and there’s one particularly memorable patient in my mind that I saw while he was at home as part of his visit with one of the medical oncologists at [name of organization]. So, the three of us had a three-way conversation. So, we set up interdisciplinary, interinstitutional care for that patient because he’s a snowbird and winters down here and summers up there and so we shared his care. That went remarkably well.

#### Care Coordination With Informal Caregivers

Participants described how telehealth made it easier to coordinate information with multiple informal caregivers (eg, inviting family members to a visit to go over a patient’s prognosis). One physician said the following:

We used Zoom so the whole family could be there. I was able to share my screen, demonstrating the tumor. They thought that was just the greatest. And, so everybody was able to be together for all that information.

Participants also used telehealth to enhance in-person visits, such as calling or videoconferencing caregivers who could not participate in the in-person visit due to on-site guest restrictions during COVID-19. A physician recalled the following:

It allowed them to have their family with them when we went over the results. Because, right now, we’re still not allowing family in the outpatient clinic. And, so it allowed our patients to be with their families so that they can ask questions and hear everything at the same time.

### Theme 3: Adapting In-Person Assessments for Telehealth

#### Overview

Participants identified challenges with patient assessments (eg, obtaining vital signs, patient-reported outcomes [PROs], and images) and conducting physical exams during telehealth visits (Table 4).

**Table 4:** Theme 3: adapting in-person assessments for telehealth codebook.

Parent code and child codes		Code definition	Code frequency across participants (N=40), n (%)
**Lack of physical exam**			
	Inability to examine lymph nodes	Apply code when participant describes the inability to feel, measure, or inspect a patient’s lymph nodes during a telehealth visit.	12 (30)
	Missing a clinical problem	Apply code when participant describes concerns over missing a clinical problem because of the inability to visualize the patient during a telehealth visit.	26 (65)
	Getting the patient involved in the exam	Apply code when participants describe strategies for getting the patient to help with the physical exam during the telehealth visit.	16 (40)
**Lack of data**			
	Images	Apply code when participants describe challenges with image resolution during telehealth visits to visualize condition (eg, wound monitoring).	15 (38)
	Vital signs and other biometrics	Apply code when participant describes not having access to vital sign or other biometric data (eg, blood pressure) that is relevant for clinical decision-making.	22 (55)
	Patient-reported outcomes	Apply code when participant describes not having access to patient-reported outcomes as a barrier for telehealth visit delivery.	11 (28)

#### Vital Sign Collection

Some participants lacked data due to scheduling challenges (eg, having a virtual visit scheduled before imaging data were available) or lack of remote monitoring (ie, ability to gather data between in-person visits). A few participants described how some patients would obtain their own vital sign data (eg, from wearable devices) and report it during the visit. As an example, one APP said the following:

It would be helpful to have vitals. I have one patient who does this by themselves, checking their own vitals (heart rate, oxygen saturation) and their blood pressure and they share it during the visit, which is great.

#### Patient-Reported Outcomes

Some participants described how it was challenging to deliver certain types of care (eg, supportive care) virtually without access to PROs (eg, depression symptoms). One psychologist explained as follows:

We used to collect the ESAS [Edmonton Symptom Assessment System] [37] for patients in person, but now, if we see a patient virtually, we don’t have that information. For supportive care, monitoring depressive symptoms is really important and now we don’t have that. We talked about having nurses call the patient to collect it over the phone before the virtual visit, but that is really not the same as getting the symptoms from the patient perspective.

During COVID-19, some clinics suspended in-person collection of PROs and did not have a means for collecting PROs as a part of virtual care. Participants also described how image quality could be challenging, requiring HPPs to follow up and obtain images after the visit. One physician indicated the following:

Some patients might have a limitation in the internet provider bandwidth, so the image resolution is poor quality. In that circumstance, what I do is I tell the patient, “Please take a picture of your surgical site with your phone, and email it to me.” But it would be nice if there was a more systematic way to do this so I could see the image during the visit.

#### Image Collection

Participants felt that most patients were comfortable sharing images with HPPs digitally and did not cite concerns about privacy. A few HPPs noted that patients over 65 years of age were more reluctant to share images due to privacy concerns compared to younger patients. One APP said the following:

Our clinic sees a lot of older patients [over 65 years] who tend to have more privacy issues with sharing images than our younger patients.

#### Physical Exams

Participants were also concerned about missing important clinical problems due to the lack of a physical exam (eg, checking lymph nodes). One APP said the following:

I really do think there’s something lost, the personal touch and the things you see with your eyes and a physical exam for a cancer patient is very important. To feel for lymph adenopathy and wherever they say their cancer was, the nearest lymph node drainage. I mean, there’s no way that I could discern that over telemedicine.

Similarly, a physician explained as follows:

I see things on my patients all the time when I see them in person. I’m like, “You need to go get that little thing on your arm there checked.” It might be a new skin lesion that they need to have looked at. I worry about missing things during virtual visits when I can’t really visualize the patient.

Some participants described getting the patient to help with conducting the physical exam (eg, having the patient measure a visible tumor) during the telehealth visit to overcome this limitation. For example, one APP shared the following:

Sometimes, I’ve had patients who’ve had visible tumors on their neck or visible tumors, I can see it on telemedicine, but I can’t really measure it. So I ask the patient to go get a tape measure and measure it.

### Theme 4: Developing Workflows and Allocating Resources

#### Overview

Participants described the importance of developing workflows for telehealth visits that are equivalent to in-person visits (eg, check-in process, EHR integration, and scheduling) and ensure sufficient resources are allocated for HPPs and patients (Table 5).

**Table 5:** Theme 4: developing workflows and allocating resources for telehealth codebook.

Parent code and child codes		Code definition	Code frequency across participants (N=40), n (%)
**Workflow**			
	Check-in process	Apply code when participant describes the check-in process used during telehealth visits.	28 (70)
	Scheduling	Apply code when participants describe how telehealth visits are scheduled (eg, batching visits).	33 (83)
	Electronic health record (EHR) integration	Apply code when participant describes lack of Zoom EHR integration (eg, inability to find visit in EHR).	16 (40)
**Resources**			
	Equipment	Apply code when participants describe equipment necessary for telehealth delivery (eg, cameras).	35 (88)
	Space	Apply code when participants describe space where telehealth visit is conducted.	36 (90)
	Clerical support	Apply code when participants describe the amount of administrative support available for telehealth visit delivery.	26 (65)
	Information technology (IT) support for patients with low digital literacy	Apply code when participants discuss IT support for patients who may have low computer or mobile health literacy.	27 (68)
	Tools for patients with disabilities	Apply code when participants discuss need for tools for patients with disabilities (eg, closed captioning and speech-to-text tools).	30 (75)

#### Check-in Process

Participants, for example, felt that the patient check-in process, which is usually handled by staff, was left up to the HPP for telehealth visits, creating inefficiency. One physician described the following:

I really am concerned about the way we’ve implemented the virtual visits is we’ve cut the nurse and the PAR [Patient Access Representative] team out of the equation so now I’m having to check in my own patients when they show up on a Zoom visit. That becomes a little more cumbersome because any time you ask me to do an administrative task, the chance that I’m gonna execute it accurately is less than if you have a nurse doing it.

#### EHR Integration

Participants also found it challenging that telehealth visits were not integrated within the EHR, making it difficult to ensure that all the HPPs involved in preparing a visit (eg, nurse and APP) could access the visit link. One APP mentioned the following:

I have great nurses who work with me and they prep the clinics and they don’t have the Zoom invitation. So, the invitation comes to me, but it doesn’t go to the nurses working with me, which makes it hard for them to prep the visit. But if somehow, the Zoom meetings were discoverable in the EHR then that would really help.

#### Scheduling

Some participants described refining their schedule to ensure that telehealth visits were grouped together rather than interspersed between in-person visits. Having telehealth and in-person visits mixed together created problems, such as forcing HPPs to run back and forth between their clinic and office where telehealth visits were conducted. One physician mentioned the following:

So, for a month or two I was running around like a chicken without a head. And finally, I said, “Enough. We’re gonna have designated days where we’re doing all the virtual visits.” So, I can sit here and do one patient after the other because it was not working, running back and forth between clinics and my office where I take the virtual visits.

#### HPP Resources

Some participants described lacking sufficient resources for telehealth visits, including equipment (eg, cameras and headphones), space (eg, finding a private or well-lit space), and administrative support. One social worker shared the following:

How are we supposed to do virtual visits when we don’t have a camera? Here we are, five or six months into this pandemic and most of us have no access to a camera outside of our cellphones.

Participants also felt that the amount of administrative support for telehealth visits was inferior to in-person visits and, as a result, much of the administrative work fell on APPs and nurses. One APP mentioned the following:

Right now, our nurse is looking ahead at who’s scheduled and making sure everything is there for the visit. But, it’s almost like if there could be someone on the back end—like they do for new patient visits that are in person—doing that, it would help the nurses. It ends up being a lot of clerical work for the nurses, taking them away from patient education.

#### Patient Resources

Most participants felt that there were sufficient resources for patients, such as the interpreter service and IT support. Participants felt that the level of IT support worked for the majority of patients but was not sufficient for patients with low mobile health (mHealth) literacy or those with a lower ability to use mHealth apps with efficiency to accomplish a task [38]. As a result, some HPPs spent a significant amount of time helping their patients with the Zoom app during telehealth visits or converted telehealth visits to virtual check-ins by phone. One pharmacist described the following:

The patients that struggled the most were patients who had to access the telehealth visit from their phone. They didn’t know how to download or locate the app. We need to make sure the training that patients receive covers how to use the app so that we don’t have to spend so much time on this during the visit.

Participants also indicated a need for more resources for patients with disabilities that may interfere with technology use. One psychologist described the following:

It was really challenging to meet virtually with patients who had trouble with hearing. I ended up converting those visits [to in person].

Similarly, a physician described the difficulty of delivering telehealth visits to patients with speech impairments, as follows:

We see patients who may not be able to talk after surgery. We would try to get the caregiver on or use the chat feature, but if they don’t have help at home, we’d have to bring them in.

### Theme 5: Recommendations for Telehealth Implementation in the Future

#### Patient-Level Recommendations

Nearly all participants were supportive of continuing telehealth beyond COVID-19 but recommended changes to ensure implementation is sustainable (Table 6). Participants provided recommendations at the patient, HPP, and organizational levels for improving telehealth implementation in the future. At the patient level, participants discussed the importance of overcoming the digital divide and recommended real-time IT support for patients, closed captioning for patients with communication-related disabilities, and educational materials on how to prepare for a telehealth visit (eg, finding a place that is comfortable). For example, one psychologist suggested the following:

I would have said a bit more patient training, just some training on not just using Zoom, but telemedicine in general, and sort of optimizing the whole visit because I’m sure there are ways to do it. For example, encouraging them to find a comfortable space, have what they need on hand.

**Table 6:** Recommendations for future telehealth implementation codebook.

Parent code and child codes			Code definition		Code frequency across participants (N=40), n (%)
**Patient-level recommendations**					
	Greater telehealth accessibility	Apply code when participants recommend strategies for improving the accessibility of telehealth (eg, closed captioning).		31 (78)	
	Real-time, information technology support	Apply code when participants recommend strategies to deliver more timely technology support to patients.		12 (30)	
	Patient education	Apply code when participants recommend strategies to deliver more patient education on how to use telehealth or prepare for telehealth visits.		23 (58)	
**HPP^a^-level recommendations**					
	Sharing information about telehealth policy changes	Apply code when participants recommend strategies to promote discussion about ongoing telehealth policy changes (eg, licensure).		21 (53)	
	Sharing best practices	Apply code when participants recommend strategies to promote discussion about telehealth best practices (eg, tips for patient engagement and checklists).		33 (83)	
	“Webside manner” training	Apply code when participants recommend HPP-level training to engage patients in a telehealth environment (eg, how to maintain eye contact).		32 (80)	
	Production support	Apply code when participants recommend support needed to professionalize the telehealth visit (eg, background and lighting).		26 (65)	
**Organizational-level recommendations**					
	Optimizing workflow	Apply code when participants recommend strategies for optimizing workflow (eg, virtual waiting room and batching telehealth visits).		30 (75)	
	Policy advocacy	Apply code when participants recommend organizational strategies for policy advocacy (eg, being more engaged with advocacy organizations).		10 (25)	
	Long-term planning	Apply code when participants recommend long-term planning strategies, such as how telehealth will be evaluated and how it will fit with other organizational priorities.		20 (50)	

^a^HPP: health care provider and professional.

#### HPP-Level Recommendations

Participants thought that HPPs should have more training on engaging patients in a virtual environment (eg, optimal eye contact and communication strategies to create a dialogue). One APP mentioned the following:

I think more training would be helpful. Not on the technology, but what is the best way to maintain eye contact with patients, how do you keep patients engaged and talking with you.

Participants also wanted more discussion about best practices in telehealth use and updates on policy changes (eg, Medicare reimbursement). One physician said the following:

I wish there was more ongoing communication, not just deliver the news without an ongoing conversation. When Medicare is going to stop covering all the telemedicine visits is a question that we all have heard. It would be helpful to have a chance to discuss this.

Participants also wanted more resources to ensure that telehealth visits were delivered in a professional and consistent way across HPPs. For example, one physician shared the following:

Professionalizing it is the highest priority. Making sure the equipment is in place, that a dedicated room is in place, that the background is place, the lighting is in place. I think we should be trying to go in a direction more like a professional broadcasting company with that level of quality because I think that adds to the performance art that is at the heart of a lot of medicine.

#### Organizational-Level Recommendations

At the organizational level, participants suggested that Moffitt engage in telehealth policy advocacy (eg, reimbursement and out-of-state licensure). One physician recalled the following:

What we had asked that patient to do was to drive themselves across the border and to have the telemedicine visit from their car. I mean, it’s an absurdity. There’s nothing we can actionably do about that except work on our government relations team and kind of change that into the future.

Participants also had suggestions for optimizing workflow, such as having a virtual waiting room and letting patients know when an HPP is running late for a telehealth visit. For example, one APP shared the following:

When I go to my cardiologist, they have a virtual waiting room that is managed by their MAs [medical assistants]. So, I log in and the MA immediately says to me, “Hi. Glad you’re with us. The doctor is running 10 minutes behind.” In our scenario, if I’m not on time, the patients think there’s something wrong with the technology and they’ll be calling the nurse. They’ll be calling like crazy. And it’s just another thing for the nurse to have to pay attention to.

Participants also recommended developing a long-term strategy for telehealth, such as how telehealth aligns with other organizational priorities and an evaluation plan to see how telehealth affects patient outcomes and health care quality and costs. One physician shared the following:

I’d like to see a long-term vision and plans for evaluating progress. We don’t really know how it will impact patients or the care we deliver or reimbursement. We should see if it is being used to its full potential in other priority areas, like clinical trials.

## Discussion

### Principal Findings

The goal of this study was to capture oncology HPPs’ experiences with rapid implementation of telehealth during the COVID-19 pandemic. Overall, oncology HPPs saw telehealth as an integral part of health care delivery moving forward—a finding consistent with other studies [20,24]—but recommended addressing key barriers to improve sustainability. At Moffitt, we plan to present the findings of this research to our leadership team to determine how the results can inform future telehealth implementation. More broadly, our research findings also provide implications for future telehealth research and practice. For example, our findings suggest that more work is needed to overcome the digital divide and ensure that HPPs have access to the resources and data necessary to deliver high-quality care in a virtual environment (eg, vital signs). Further, the results suggest that a long-term strategy is needed to determine how telehealth will be integrated across the cancer care continuum and monitored to assess impact on patient outcomes and health care delivery. At the same time, health care systems will need to develop a research and policy agenda to ensure that evidence informs telehealth policy approaches, and that the regulatory and payment landscape accelerates and facilitates optimal telehealth use in cancer care.

### Addressing the Digital Divide

Health care systems have implemented innovative strategies to address the digital divide (ie, disparities in technology access, skills, and use) during COVID-19, such as assessing patients’ readiness for telehealth [39-42]. A key step to overcoming the digital divide is understanding which patients within a system are impacted by the digital divide and what specific barriers they experience. For example, smartphone-only internet access may bridge the digital divide for some patients, but it could limit health care access for others who have limited data plans or limited experience with mobile apps [18,43]. To address digital health literacy (ie, the ability to use computers and search for and evaluate health information electronically), some health care systems have deployed social work staff to help patients access telehealth [18,41]. In our system, we provided IT support to all patients with scheduled telehealth visits, but for some patients, more support was needed. Health care systems have also experimented with device loan programs and partnering with community-based organizations to create spaces where patients can connect to the internet [21,41,43]. The Veterans Health Administration (VHA) recently developed a partnership with a cellphone carrier to ensure patients could access VHA telehealth apps without affecting their data limits [21]. Prior studies also suggest that experience with technology is a key predictor of technology use for health care (eg, how often a patient uses the internet or a smartphone) [44]. Further studies should explore longitudinal models of technology training that move beyond a one-time training and allow for repeated experiences with technology use for health care. Further, our health care team members found that current telehealth apps may not be optimized for patients with disabilities (eg, lack of closed captioning and speech-to-text tools), similar to other studies [21,45,46]. To improve access among patients with disabilities and other conditions that could affect human-computer interaction (eg, low literacy), technology vendors should include patients who are affected by the digital divide in co-designing and testing of new products [39,46,47]. Future studies should also consider community-based participatory research approaches in the development of digital health technologies to better engage patients affected by the digital divide [48]. Community-based participatory research has been used in mHealth studies, for example, to increase community participation in app development, usability testing and app refinement, and designing recruitment, implementation, and dissemination strategies [48].

Our study participants noted that access to interpreters was a key ingredient of successful telehealth deployment. Prior studies have documented disparities in telehealth use among patients who prefer English and those who do not prefer English in the United States during the pandemic [49-52]. Similar disparities have been observed in patient portals, which are used by some health care systems to deliver telehealth [53,54]. Qualitative studies have documented barriers, such as limited access to professional interpreters, lack of bilingual HPPs, and lack of translation of COVID-19–related informational materials [55]. Additional studies are needed to better understand telehealth access among patients who do not prefer English. For example, a previous study demonstrated that factors such as interpreter modality (eg, professional vs ad hoc and video vs in person) affect the accuracy of interpretation for health care visits [56]. Future studies could compare different models of interpreter services through telehealth and compare interpretation accuracy rates and other outcomes, such as patient satisfaction. Researchers have also recommended that health care organizations develop monitoring systems for evaluating disparities in telehealth uptake by language preference (eg, dashboards), develop telehealth and patient portal trainings in multiple languages, and prioritize the hiring of bilingual HPPs [57]. More research is needed to develop and test implementation strategies that address disparities in telehealth access based on language preference.

### Ensuring HPPs Have Sufficient Tools for Telehealth Implementation

HPPs may need additional resources to deliver telehealth effectively. HPP-facing tools [58,59] may be helpful in providing education on “webside manner” and implementation checklists [58,59]. HPPs at Moffitt indicated a need for guidance on the optimal way to conduct a telehealth visit (eg, lighting and eye contact), similar to other studies [40,60]. Health care systems could disseminate available trainings (eg, Academy of Communication in Healthcare training) or develop institution-specific trainings [61]. Some health care systems have developed implementation checklists for health care team members that include helpful tips, such as confirming a patient’s phone number at the beginning of the virtual visit in case the technology fails [62]. Patient-facing tools may also improve virtual patient-HPP communication. Some HPPs have developed patient handouts on how to conduct elements of a physical exam during a telehealth visit and how to prepare for the visit (eg, patient positioning) [23]. Similar approaches could be tested more broadly. Other studies have noted that HPPs may lack sufficient digital health literacy [63,64], which can negatively affect engagement with health IT systems (eg, EHRs) [65]. This concern was not raised by our participants, but future studies should explore the effects of HPPs’ eHealth literacy on telehealth implementation.

### Remote Monitoring for Telehealth Implementation

Access to patient data during telehealth visits, such as biometric data and PROs, is also critical to implementation success. A recent study in primary care found that blood pressure assessments declined by 37% during COVID-19, in part due to lack of biometric screening during virtual visits [66,67]. These findings highlight the need for remote monitoring programs, which will require health care systems to invest in complex change (eg, EHR integration of biometric data and optimized data visualization) [68-72]. Prior to COVID-19, there was limited reimbursement for remote monitoring, hindering health care system adoption [73]. During the pandemic, Medicare has expanded payment policies for remote monitoring with certain restrictions (eg, type of data and minimum amount of data needed) [74]. Some cancer care systems have started to invest in remote monitoring programs and cited recent changes in Medicare policy as a motivator for adoption [75]. Cancer care systems will need to evaluate the effectiveness of remote monitoring programs and identify areas in which remote monitoring adds the most value. Like other studies, our research found that older patients may have more privacy concerns about remote monitoring and sharing patient-generated health data, such as images, compared to other patients [76,77]. Prior studies recommended strategies that strengthen patient activation and HPP-patient trust; they also recommended developing patient education programs about how data are being used and protected in order to overcome privacy-related barriers to sharing patient-generated data [76,77]. Other privacy-related barriers (eg, concerns about information security) were not mentioned by participants in our study but deserve consideration in future telehealth research. Organizations have reported examples of “Zoombombing,” or when an intruder joins a Zoom videoconference [78], raising concerns about information security and telehealth. Researchers have recommended that health care organizations develop multipronged approaches (eg, employee training and simulated cyberattacks) to address information security threats in telehealth [79]. Further, some have argued that health care organizations should transition from consumer-oriented videoconference tools that were adopted at the onset of the pandemic to health care–specific videoconference tools with additional security features [79]. More research is needed to identify best practices in information security for telehealth as it grows in usage.

### Greater Evidence Regarding Effectiveness, Implementation, and Potential Risks of Telehealth

Cancer care systems, payers and insurers, and policy makers will need more evidence for the effectiveness and safety of oncology telehealth to guide future decisions. Research from other health care sectors has demonstrated that telehealth can be equivalent to in-person care for certain conditions and offers a relative advantage over in-person care (eg, reducing rural health care disparities) [11,80-83]. Telehealth also has potential risks, such as inappropriate antibiotic prescribing or exacerbating existing health care disparities due to the digital divide [26,84-89]. Within oncology, telehealth models for supportive and survivorship care and ancillary services (eg, genetic counseling) have proven effective, but there is limited evaluation of telehealth for other areas of care (eg, screening, diagnosis, treatment, and surveillance) [90-95]. Therefore, it is critical to evaluate telehealth use in these areas and assess impact on patient outcomes, health care quality, costs, equity, and potential risks (eg, inappropriate care). Further, research is needed to evaluate strategies for incentivizing the use of telehealth in a postpandemic landscape [96]. One strategy may be alternative payment models, which have increased telehealth adoption in other health care sectors but are understudied in oncology [97]. Further, as adoption decisions move from mandatory to voluntary, studies should test theories of technology adoption (eg, technology acceptance model) to examine what factors help explain sustained telehealth use beyond the pandemic [98,99].

### Policy Advocacy for Telehealth

Cancer care systems and key stakeholders will need to develop an agenda to ensure that future policies are supportive of oncology telehealth. During the pandemic, many state-level restrictions were lifted that made it easier to implement telehealth, including waiving out-of-state licensure requirements or expanding payment parity [4,15]. Further, many state Medicaid programs and commercial insurers changed telehealth policies in response to COVID-19 (eg, removing cost-sharing requirements) [4,15]. At the federal level, there was a major overhaul of Medicare telehealth payment policies (eg, allowing virtual check-ins through telephone to qualify for telehealth), resulting in 244 temporary regulatory changes [4,15,100]. Uncertainty remains regarding which federal- and state-level policies will be retained in the future [4,15]. There has also been federal investment in overcoming the digital divide in the United States [101]. The Infrastructure Investment and Jobs Act became public law recently and provides funding for expanding broadband access in low-income neighborhoods, reducing practices of digital redlining (ie, limited internet service provision in low-income and high–minority concentration neighborhoods), and expanding internet subsidies for individuals with limited economic resources [101]. Future research will be needed to monitor program implementation and effectiveness for addressing digital disparities at the federal, state, and local levels. Moving forward, a policy agenda will need to include greater investment in telehealth research, addressing medical licensure and credentialing barriers [102], and a balanced approach to regulation, one that continues to fuel innovation in telehealth for oncology while safeguarding against potential risks (eg, delivering telehealth for a condition that is not “tele-amenable” [96]). For instance, among our study participants, some HPPs expressed concerns about using telehealth to deliver information about a new and serious cancer diagnosis. A recent survey among oncologists (n=29) identified similar findings: some oncologists were reluctant to use telehealth for delivering bad news [103]. Additional studies are needed to determine optimal use of telehealth in oncology to guide future policy.

### Limitations

This paper has several limitations. First, this is a qualitative study from an NCI-Designated Comprehensive Cancer Center and the findings may not be generalizable to other settings. Second, there are other health care professionals (eg, genetic counselors) who have used telehealth as a part of their practice, and their experiences were not captured in our sample. Third, we limited the interviews to 30 minutes or less to increase participation, and we reduced the number of questions included in the interview guide. Therefore, some topics, such as how HPPs have used telehealth for clinical trials, were not discussed. Fourth, our study excluded residents and fellows who were important stakeholders in telehealth implementation. Future studies should examine the unique experiences of residents and fellows who are simultaneously learning how to deliver care in person and virtually. Finally, it was beyond the scope of this research to capture the patient perspective. Studies have started to assess patient experience with telehealth during the COVID-19 pandemic; however, there has been limited study of this in oncology [104-106]. Future studies should assess the perspectives of patients with cancer regarding telehealth to explore patient satisfaction, barriers and facilitators to telehealth access, and patient preferences for telehealth (eg, whether certain services should be delivered in person vs virtually and preferences for how interpreter services should be implemented).

### Conclusions

Overall, cancer care frontline HPPs have used innovative and adaptive strategies to rapidly implement telehealth during COVID-19 and are supportive of continuing virtual cancer care delivery beyond the COVID-19 pandemic. HPPs identified several facilitators for telehealth implementation, such as improved care coordination with other HPPs and informal caregivers. HPPs also noted several barriers, such as lack of physical examinations and vital sign information, which limited HPPs’ ability to fully evaluate a patient. To support the rapid growth of oncology telehealth, implementation strategies are needed to overcome the digital divide and ensure that HPPs and patients have the tools necessary to effectively engage in telehealth. Health care systems, policy makers, health insurers, and payers must develop long-term strategies for integrating telehealth into the cancer care continuum, building the evidence base around telehealth in oncology, and developing a policy agenda that will advance telehealth innovation while safeguarding against potential risks.

## Appendix

Multimedia Appendix 1 Interview guide.

## Abbreviations

APP: advanced practice provider
CFIR: Consolidated Framework for Implementation Research
CMS: Centers for Medicare & Medicaid Services
EHR: electronic health record
ESAS: Edmonton Symptom Assessment System
HPP: health care provider and professional
IT: information technology
MA: medical assistant
mHealth: mobile health
Moffitt: Moffitt Cancer Center
NCI: National Cancer Institute
PAR: Patient Access Representative
PRO: patient-reported outcome
VHA: Veterans Health Administration
