# Supplementary material for: Health Care Providers’ and Professionals’ Experiences With Telehealth Oncology Implementation During the COVID-19 Pandemic: A Qualitative Study
Source: J Med Internet Res. 2022 Jan 19;24(1):e29635. doi: 10.2196/29635 (PMC8772877; doi:10.2196/29635)
Supplement: Multimedia Appendix 1 [file jmir_v24i1e29635_app1.docx]

**Multimedia Appendix 1. Interview guide**

| **Interview Section** | **Questions** |
| --- | --- |
| Interviewee characteristics | What is your role at the cancer center?   - PROBE: How long have you worked here? - PROBE: Do you work primarily for the cancer center or do you practice at multiple organizations? |
| Telemedicine experience prior to COVID-19 | Before COVID-19, what types of telemedicine, if any, did you provide to your patients?   - PROBE: What types of care did you deliver through telemedicine? - PROBE: What types of patients did you see through telemedicine? |
|  | How easy was the virtual visit platform to use?   - PROBE: How were you able to have the information you needed about the patient available? - PROBE: What made your use of the system productive or challenging? |
| Telemedicine use during COVID-19 | With the onset of COVID-19, are you now providing telemedicine to your patients?   - PROBE: What types of visits? For which types of patients? (new encounters? Follow up?) - PROBE: Has your patient load been heavier or lighter or about the same? - PROBE: What systems did you use to provide telemedicine How easy is this system to use? How productive are you able to be with this system? |
| Access to information about telemedicine | How helpful is the information provided by the cancer center around telemedicine?   - PROBE: What additional information would have been helpful for you to have had on your patients when you see them virtually or by phone? - PROBE: Did you receive any training or other support/resources/polices that were helpful as telemedicine was implemented? - PROBE: Are there resources or other things that would have been helpful? |
| Adaptations | What kinds of changes or alterations to care delivery did you have to make to shift from in-person to virtual care?   - PROBE: Are there additional things you have added or taken away from your typical patient care visits? - PROBE: Are there additional things you wish you could supplement a telemedicine visit with (e.g., remote patient monitoring, home visits)? |
| Barriers | Are there things that make it challenging for you to deliver telemedicine?   - PROBE: Thinking about the entire visit continuum, from pre-visit prep to after visit follow up, which aspects of care have been most challenging to deliver via telemedicine? |
| Patient-physician and informal caregiver interaction | How has telemedicine changed the way you interact with your patients?   - PROBE: How, if at all, has the use of telemedicine improved your interactions with patients? - PROBE: Has it improved or hurt your interactions with patients? - PROBE: Has coordination with family members and caregivers changed? If so, in what way? |
| Patient acceptability | How have your patients responded to telemedicine? Are there subgroups of your patients for whom telemedicine has worked well? Been more challenging?   - PROBE: What strategies have you used to help patients overcome barriers to using telemedicine? - PROBE: Have your patients’ compliance with treatment changed in any way due to telemedicine? |
| Care coordination | How has telemedicine changed the way you interact with other healthcare providers?   - PROBE: How has the use of telemedicine impacted care coordination? |
| Additional resources | Are there any changes needed, such as additional resources, for you to be able to deliver telemedicine effectively?   - PROBE: For example, is there additional computing resources, staff support, or office space you need? Additional pre- or post-visit support needed? Something else? |
| Supportive of future use | Are you supportive of continuing to provide telemedicine to your patients in the future? Why or why not?   - PROBE: If they are not sure, ask them what information they’d like to have to help them form an opinion about telemedicine. |
| Services best suited for telemedicine | What types of visits work best with telemedicine? What types of visits do not work well with telemedicine? |
| Lessons learned and recommendations | Do you have any lessons learned or recommendations you would share on how to improve telemedicine implementation in the future? |
